# Perineuronal nets in the developing brain: implications for neurodevelopmental disorders

**DOI:** 10.1186/s13041-025-01261-3

**Published:** 2025-11-24

**Authors:** Jennifer M. Ackerman, Thomas James L. Ford, Shraddha Shridhar Kattewar, Woo-Yang Kim

**Affiliations:** 1 Department of Biomedical Sciences, Kent State University, Kent, OH USA; 2 Department of Biological Sciences, Kent State University, Kent, OH USA

**Keywords:** Brain development, Perineuronal nets, Extracellular matrix, Synaptic plasticity, Neurogenesis

## Abstract

Here, we review recent findings on the development, functions, and alterations of perineuronal nets (PNNs) in relation to neurodevelopmental pathologies. PNNs are dense extracellular matrix structures primarily found in the central nervous system, comprising a heterogeneous array of components surrounding neurons. They play a crucial role in neuronal maturation and function, particularly in synapse formation and stabilization, which impacts higher-order brain connectivity. Emerging evidence underscores the dynamic changes in PNN composition and distribution during neuronal plasticity, with PNN remodeling shown to influence social and cognitive behaviors such as learning and memory. Conversely, disruptions in PNN dynamics have been implicated in developmental brain disorders. This review aims to present recent advancements in PNN neurobiology and to integrate these findings into our understanding of the mechanisms underlying neurodevelopmental pathogenesis.

## Introduction

The extracellular matrix (ECM) is a mosaic of various non-cellular macromolecules that are tissue or cell-specific throughout the entire body [152]. The ECM surrounding cells in the brain provides both structural and functional support. Within the brain, the ECM constitutes approximately 20% of the mature adult brain, while it consists of up to 40% of the developing brain [130]. In 1893, Camilo Golgi first described a condensed form of the ECM that enwrapped cell bodies and proximal dendrites, now known as perineuronal nets (PNN) [44]. PNNs have long been considered one of two specialized forms of the ECM in the central nervous system, with the other specific form being the basement membrane [123]. However, PNNs have recently been found in the peripheral nervous system, although the scope of their presence needs further investigation [1].

PNNs are specialized structures that surround populations of metabolically active interneurons, specifically those found in the central nervous system [73, 81, 104, 149]. Historically, it was believed that they restricted synaptic plasticity solely in adulthood, with PNNs emerging postnatally, coinciding with the end of critical periods of plasticity [19, 59]. However, more recent studies have found that PNNs have a larger role within neural circuits. Their emergence around metabolically active parvalbumin-positive interneurons (PV) may help to protect these highly active interneurons from oxidative stress allowing them to preserve their structural connectivity [18, 103]. Furthermore, recent evidence has emerged that PNN remodeling may also be essential during active learning [10, 90, 145]. This suggests that PNNs are not merely static in adulthood, but continue to shape connectivity far into adulthood.

Recently, PNNs have further emerged as essential components in understanding the pathology of neurodevelopmental and neuropsychiatric disorders, as their dynamic role in adulthood has been elucidated. Due to their regionality and vulnerability to stress, alterations to PNN densities affect a range of behaviors in various ways [6, 107, 131, 133]. These findings have sparked a recent interest in PNN-targeting therapies to enhance cognitive and emotional function by modulating plasticity specifically.

This review examines the intricate development of PNNs and their functional implications in the mammalian brain, with a particular focus on their diverse molecular composition. We examine how PNNs contribute to critical neurobiological processes, including synaptic stabilization, neuronal signaling, and the closure of critical periods of plasticity. Additionally, we highlight the broad and growing recognition of PNN dysfunction as a contributing factor in a range of neurodevelopmental and neuropsychiatric disorders, underscoring its potential as a therapeutic target in the treatment of these conditions.

### PNN structures and components

**Fig. 1:**
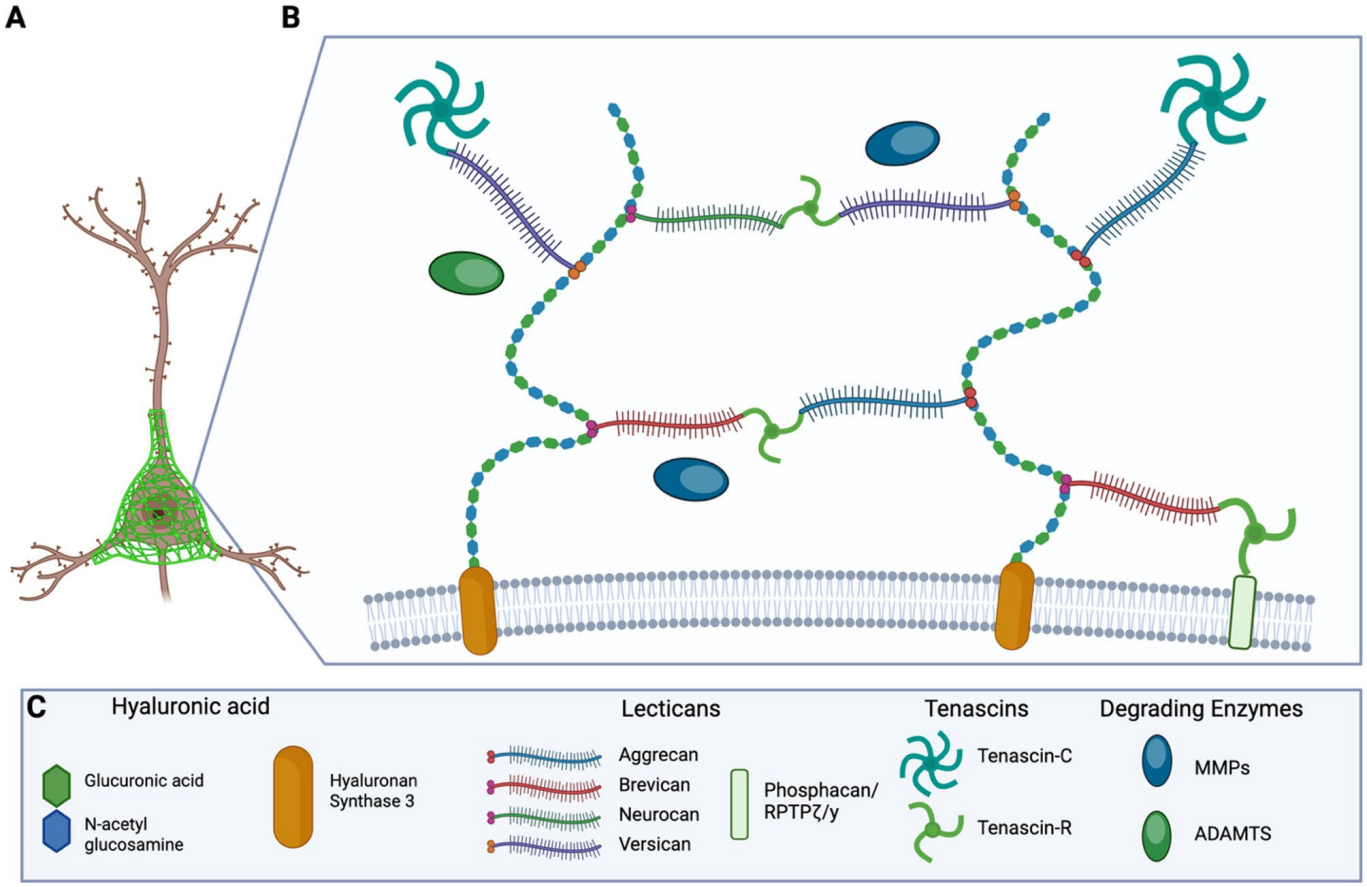
Graphical representation of a PNN surrounding a neuron, and the structural composition of a PNN. **A** Representation of the PNN surrounding a cortical interneuron. **B** Diagram of a simplified PNN and its constituents. **C** Graphical representation of the various molecular components used above (Created with Biorender.com).

Despite being extracellular matrix structures, PNNs display a highly consistent architecture. At the core of this structure are repeating units of hyaluronic acid (HA), which form the primary scaffold of the PNN (Fig. 1). HA is synthesized by hyaluronan synthase 3 (HAS3), a transmembrane protein expressed in neurons. This HA scaffold facilitates the attachment of lectins through the action of link proteins, primarily hyaluronan, and proteoglycan link proteins 1 and 4 (HAPLN 1/4), which bind to the globular domains at the N-terminal of lecticans, stabilizing the PNN structure [91].

Within PNNs, there are four types of known cartilage-specific proteoglycan core proteins (CSPCP) that belong to the lectican family and are distinguished by differences in their core protein size and the number and composition of GAG chains: Aggrecan (ACAN), Brevican (BCAN), Neurocan (NCAN), and Veriscan (VCAN). ACAN is the most extensively studied lectican and is secreted by neurons and astrocytes [110]. While neurons also secrete BCAN, NCAN, and VCAN, their secretion is dependent mainly on glial cells [42]. *Wisteria floribunda* agglutinin (WFA) is the earliest and most common PNN marker. It binds specifically to the N-acetyl-galactosamine residues on the GAG repeats of the four lectins [47].

Tenascin-R (TnR) serves as a key linking protein that connects lectican to HA chains within PNNs (Fig. 1). It is exclusively secreted by oligodendrocytes and neurons postnatally [98]. TnR binds to the C-terminal globular domain of lecticans in a calcium-dependent manner [2, 34, 114]. TnR-deficient mouse models have shown disrupted ACAN clustering. This defect can be rescued by the application of exogenous TnR, demonstrating TnR’s essential role in net stabilization [85]. Tenascin-C (TnC) appears to have a more regulatory role, interacting with the environment outside of the PNN (Fig. 1). TnC is secreted primarily by astrocytes and radial glial cells. However, it is also produced by specific neuronal populations, including granule cells in the dentate gyrus, motor neurons in the spinal cord, and pyramidal cells in the cerebral cortex [4, 9]. Compared to TnR, TnC is expressed at relatively low levels in cortical areas rich in WFA-positive PNNs, suggesting that TnC is not an essential structural component of PNNs [53]. This is supported by TnC-deficient mouse models, in which PNN formation within the hippocampus appears to be unaffected. However, long-term potentiation in the hippocampal CA1 was reduced, indicating an essential role for TnC in synaptic plasticity rather than net assembly [33]. Furthermore, these mouse models have exhibited behavioral abnormalities, impaired locomotion, and reduced levels of serotonin and dopamine in the cerebral cortex, hippocampus, and striatum [39, 86].

### PNN formation and maturation in the developing brain

Surrounding developing PV-positive interneurons, PNNs form through the condensation of chondroitin sulfate proteoglycans (CSPGs) [73, 81, 104, 149]. Notably, the sulfation patterns of the GAGs, precisely the 4-sulfation/6-sulfation ratio, are critical not only for proper PNN development but also for the function of PV interneurons [80, 81]. These sulfation patterns have been shown to influence the activity of orthodenticle homeobox 2 (Otx2), a homeoprotein essential for the maturation of PV neurons [126]. Otx2 in PV neurons regulate both the onset and the closure of the critical period of plasticity. For example, an experience-dependent increase in Otx2 within the visual cortex contributes to the termination of the critical period, leading to a subsequent reduction in neural plasticity [81, 126].

It is essential to note that while PNNs closely interact with glial cells, successful PNN development has been observed in cell cultures that lack glia [82]. Although early data showed that some PNN components are produced by glial cells, these components are still present in the absence of glia, suggesting that glial input is not essential for PNN formation. Instead, the neurons that PNNs wrap around can develop independently, synthesizing and secreting the necessary components for net assembly [20, 21, 40].

Unlike many structures in the central nervous system, PNNs exhibit a relatively late developmental onset [31]. This delayed critical period of development is particularly significant because degradation of PNNs at this stage can return the affected brain region to a more developmentally plastic state [19, 31, 81, 99]. Notably, this critical period has been found to be sex dependent. For instance, female rats display earlier PNN maturation in the medial prefrontal cortex (mPFC) [31]. The critical period refers to the transition marked by a decline in neuronal plasticity and the establishment of mature synaptic signaling in neurons surrounded by PNNs [81, 109]. The timing of the critical period for PNN development varies across brain regions, with no consistent age of onset or maturation [136]. The visual system, for example, has been hypothesized to be dependent on sensory experience and typically begins around postnatal day 14 (P14) in mice [81, 149]. Maturation of PNNs in the visual cortex is generally complete between P30 and P45 [149]. Furthermore, even within the cortex, considerable variation exists in the timing of PNN development across different subregions [136]. While the visual system provides a clear model of experience-dependent PNN maturation, this pattern is not generalized to all brain areas. Various brain regions exhibit distinct timelines of the onset, development, and maturation of PNNs as summarized in Table 1.

**Table 1:** Onset, development, and maturation of PNNs in various brain regions

Region	Onset	Maturation	References
Visual cortex (V1)	P10-28	P30-45 (Plateau at P42)	[149]
Visual cortex (V2, V3)	P10-14 No earlier ages observed	P25-29 No decline at P60-70 No other ages observed	[23]
Visual cortex (V5)	P10-14 (No earlier ages observed)	P25-29 Slight decline at P60-70 No other ages observed	[23]
Prefrontal cortex (PFC) layers 2/3 and 5/6	P15-22	P22-60	[127]
Hippocampus (CA2 highest density)	P10-25	P25-50	[17]
Basolateral amygdala	P16-28	P28-P50	[43]
Medial entorhinal cortex	P12-17	P17-P30	[64]
Somatosensory cortex	P9-P21	P21-P56	[135] [75]

PNN development in the sensory cortex is highly dependent on external stimuli [135, 149]. More broadly, the formation and maturation of PNNs rely on both intrinsic neuronal activity and sensory-driven input from the surrounding environment [111]. In the sensory system specifically, external stimuli have been shown to directly influence PNN development in the visual, auditory, and somatosensory cortices [88, 135, 149]. Across multiple studies, sensory deprivation has been shown to lead to underdeveloped PNNs in the affected sensory regions, underscoring the critical role of environmental input in shaping PNN maturation [111].

Although PNNs are believed to reach a mature status around 8–12 years of age in humans, multiple studies have found that PNN density continues to increase with age up to 84 years old [63, 78, 109]. This increase in PNN density with age is also seen in rodents within the inferior colliculus and sensory cortex [74, 136]. This increase in PNN density is likely due to changes in the sulphation patterns in the ageing brain. Chondroitin 6-sulphates are more plastic and associated with the developing brain, while chondroitin 4-sulphates are inhibitory, with the balance between them regulating plasticity [67, 81, 140]. The sulphation pattern in the brain changes with age, with nearly all C6S dissipating after 20 months in rodent models [38, 148]. Elevation of PNN density may restrict synaptic plasticity by preventing synaptogenesis, resulting in age-related cognitive impairments [35].

### Distribution patterns of PNNs in the brain

According to a comprehensive atlas, PNNs are highly expressed in the neocortex, hippocampus, diencephalon, and brainstem. In the majority of brain regions, only 20%-30% of PV-positive interneurons are enwrapped by PNNs. However, within the neocortex, hippocampus, and striatum, colocalization is significantly higher, averaging 40–50% and upwards of 70% colocalization in specific subregions [73]. A high distribution of PV-positive PNNs spans cortical layers 2 through 6. Among these, layer 4 exhibits the highest PNN density at P42, while layers 2/3 and 6 show comparatively lower densities [149]. Cortical layers 5 and 6 display the highest colocalization between PV neurons and PNNs [149]. Uniquely, within the hippocampal area CA2, PNNs surround excitatory pyramidal neurons. Additionally, these pyramidal neurons express mRNA transcriptions for the secretion of ACAN, suggesting that these neurons are a novel source of PNN in the hippocampus [17].

Within the cerebellum, PNNs are most prevalent in the deep cerebellar nuclei, enwrapping greater than 90% of the neurons. Whereas within the cerebellar cortex, PNNs enwrap fewer than 10% of neurons [87]. PNN densities within the cerebellar cortex are most intense in the granular layer, with prominent PNNs surrounding inhibitory Golgi cells [25]. However, the lugaro/gloubular cells of the granular layer and Purkinje layer are also enwrapped in PNNs [26]. Terminal branches of the Purkinje axon within the infraganglionic plexus of the granular layer have clear PNNs; however, the aligned Purkinje cell bodies exhibit minimal enwrapment by PNNs. Notably, within the molecular layer, WFA staining is minimal and nonspecific, indicating a lack of any substantial PNN density [25].

Despite the long-held belief that PNNs are exclusive to the central nervous system (CNS), recent findings have identified their presence within the peripheral nervous system (PNS) as well. Notably, deficiencies in Tenascin-X (TnX), which is an extracellular matrix glycoprotein structurally similar to TnR and TnC, have been associated with functional impairments in enteric motor and nociceptive sensory neurons in the PNS [1]. Further examination has revealed the presence of PNNs in spinal afferents of the distal colon. Additionally, there is an increase in PNN-related components in a mouse model of ulcerative colitis, suggesting a broader role of PNNs than previously thought [27].

### Role of PNNs in neuronal protection and synaptic plasticity

PNNs have been traditionally associated with the restriction of synaptic connectivity, serving as a physical barrier for synaptic connections. Enzymatic degradation of PNNs has been shown to increase the number of synaptic connections, supporting their role in suppressing synaptic connectivity [65, 101, 121]. Furthermore, CSPSs, have been shown to inhibit neurite outgrowth for plasticity regulation [19, 59]. However, more recent studies suggest that PNNs may also play a protective role and exhibit a degree of plasticity even in the adult brain [10, 13, 18, 90, 103, 145].

PV interneurons are particularly susceptible to oxidative stress due to their increased mitochondrial activity, resulting from their rapid firing properties [48, 55]. PNNs have been shown to protect PV interneurons from oxidative stress. For instance, PV interneurons lacking PNN exhibit elevated levels of 8-hydroxy-2’-deoxyguanosine (8-oxo-dG), a biomarker of oxidative stress, and increased apoptosis in a mouse model of schizophrenia [13, 28]. Further investigations have demonstrated that enzymatic degradation of PNNs using chABC renders PV interneurons more vulnerable to oxidative stress, suggesting the neuroprotective role of the CS-side chains of CSPGs [7, 13]. Supporting this, chABC has been shown to activate multiple protein kinase pathways, including protein kinase C (PKC) and phosphatidylinositol 3-kinase/protein kinase B (PI3K/Akt), which promote the expression of the antioxidant enzyme heme oxygenase-1 [14, 146]. PNNs may also act as a physical barrier against oxidative stress-inducing molecules, such as metal ions, which catalyze the formation of free radicals through reactions with cellular metabolic byproducts, contributing to neuronal apoptosis. Additionally, neurons lacking PNNs display accumulation of lipofuscin, a byproduct of iron-catalyzed oxidative stress, further suggesting their increased susceptibility to oxidative stress and apoptosis [84, 128, 129]. PNNs also seem to have a protective role against neurodegeneration, such as Alzheimer’s Disease (AD). Cortical regions with high amounts of CSPGs are less vulnerable to AD-related degradation by amyloid plaques [12, 83].

Beyond their roles in limiting synaptic connectivity and protecting neurons from oxidative stress, PNNs also exhibit plasticity in the adult brain, extending the closure of the critical period. The closure of the critical period, whose timing varies across the brain regions, marks the end of juvenile plasticity and establishes the proper balance of excitation and inhibition within neural circuits [102]. However, accumulating evidence suggests that adult PNNs remain plastic in adulthood and may play an essential role in learning and memory. Exogenous enzymatic degradation of PNNs using chABC has been shown to reintroduce juvenile-like plasticity and improve cognitive functions, including learning and memory [49, 147]. However, it is also important to note that intact PNNs are necessary for memory retention. While degradation of PNNs via chABC enhances acquisition of learning tasks such as eyeblink conditioning, it has been associated with impaired memory retention. These findings suggest that PNNs must be dynamically plastic, capable of remodeling while retaining their structural integrity to support optimal memory formation and maintenance [18].

Microglia are immune cells in the central nervous system that regulate the CNS microenvironment through numerous mechanisms, including the secretion of matrix metalloproteinases (MMPs) [24, 141]. MMPs are endogenous proteolytic enzymes that have been implicated in PNN remodeling by digesting CSPGs [22, 37, 125]. Recent research has found a significant negative correlation between the density of activated microglia and PNN density within the hippocampus and visual cortex, suggesting a direct role between microglial activity and PNN remodeling [68]. These findings are supported by previous research showing that the elimination of microglia prevents PNN loss via colony-stimulating factor 1 receptor (CSF1R) inhibition [3]; Liu et al. [70]. Furthermore, ex vivo imaging studies have shown that microglia active by repeated ketamine exposure are in constant contact with the soma of PNN-enwrapped neurons and accumulate WFA-positive fragments within their soma [139]. Notably, MMP-9 expression is elevated not only by immune responses, but also by inhibitory avoidance and spatial learning tasks in adult rodents, supporting the idea that PNNs are plastic during periods of active learning [10, 90, 145]. Furthermore, inhibition of MMPs has been shown to disrupt the reconsolidation of fear memories, suggesting that PNN remodeling is involved in memory reconsolidation processes [11].

Astrocytes are star-shaped glial cells that provide both structural and metabolic support to the CNS, while also regulating extracellular homeostasis [144]. Astrocytes play a crucial, yet indirect, role in the formation and modulation of PNNs. Immature astrocytes increase connexin levels, leading to the activation of the RhoA-Rock pathway, which in turn suppresses MMP-9 and facilitates the condensation of PNNs during the critical period [106]. As discussed earlier, many components of PNNs are produced by astrocytes; however, their role in maintaining adult PNNs in homeostatic conditions remains unclear [112]. When astrocytes are transformed into active astrocytes in response to disease and injury, that degrade CSPGS via the release of a disintegrin and metalloproteinase with thrombospondin motifs (ADAMTS) proteinases [29]. Glioma-associated epilepsy, PNN disintegration was not correlated with reactive astrocytes, suggesting that astrocytes may play a more indirect role in PNN maintenance that is yet to be fully established [132].

PNNs have also been shown to follow a diurnal rhythm, with PNN density decreasing during periods of sleep, in both humans and mice. Interestingly, sleep deprivation prevents sleep-associated reduction in PNN density and enhances fear memory extinction in adult rodents [94]. These findings suggest that the decrease in PNN density during sleep may facilitate synaptic reorganization, allowing for the dynamic regulation of synaptic plasticity in the adult brain, as discussed above. Further research on the diurnal rhythms of PNN has found that an increase in PNN density precedes changes in 8-oxo-dG intensity. This temporal relationship suggests that PNNs may be upregulated in anticipation of increased metabolic activity, suggesting that their neuroprotective effects are also dynamic in a time-dependent manner [46]. Together, these findings underscore the multifaceted and dynamic roles of PNNs in maintaining neural function and plasticity across the sleep-wake cycle.

### Impact of PNNs on cognition, social, and emotional behavior

The behavioral effects of PNNs are wide-ranging, primarily due to their broad presence throughout the brain, as described previously, and their direct role in regulating synaptic connectivity. PNNs play a crucial role in shaping neural circuitry, and their influence on synaptic plasticity contributes to a range of behavioral outcomes. A growing body of evidence indicates that PNNs are involved in the pathophysiology of numerous neurodevelopmental and neuropsychiatric disorders. Moreover, PNN structure and function are sensitive to stress across the lifespan, from early development through adulthood, further emphasizing their importance in maintaining neural and behavioral health.

The dynamic remodeling of PNNs is essential not only for the formation and maintenance of memories but also adaptive application of learned knowledge. Enzymatic degradation of PNNs, particularly through chABC, has been shown to improve memory performance, likely due to increased synaptic connectivity and plasticity [49, 147]. Yet the findings from degradation studies also indicate that PNNs are critical for memory retention, highlighting a complex dual role in both facilitating and stabilizing memory processes [18]. Additionally, chABC-mediated degradation of PNN promotes the elimination of fear memories, further supporting the idea that PNNs are essential for forming strong, long-lasting memories [43]. Furthermore, the timing of PNN degradation appears to be crucial. Administration of chABC prior to fear memory acquisition in adult mice increases fear extinction in juvenile rats. In contrast, injection after fear conditioning but before extinction did not significantly affect extinction outcomes. These results suggest that PNNs are primarily involved in the encoding and retention phases of memories, rather than in the direct elimination of previously acquired memories [89].

The role of PNNs in social behaviors appears to be more explicit, although current findings remain relatively limited. Within multiple models of autism spectrum disorder (ASD), overexpression of PNNs has been consistently associated with social interaction defects in adulthood, such as reduced preference for social novelty [41, 77]. Furthermore, application of chABC in the PFC of a *CNTNAP2* knockout mouse model of ASD rescued social interaction deficits, although it did not affect restricted or repetitive behaviors [41]. While in a model of adolescent intermittent ethanol exposure (AIE), PNN levels are dynamic and are correlated with social impairments in male rats only. In adolescent males, AIE initially decreases PNNs in the prelimbic cortex, followed by a compensatory increase in adulthood [134]. These findings suggest that PNN alterations during critical developmental windows may contribute to long-term deficits in social behavior in a potentially sex-dependent manner.

In both humans and rodents, PNNs are sensitive to early-life stress (ELS), particularly during the juvenile and adolescent developmental periods. The effects of ELS seem to be highly dependent on the specific stress paradigm, the timing of exposure, and sex. For instance, perinatal exposure to the antidepressant fluoxetine has been associated with decreased PNN density in the basolateral amygdala and hippocampus in both juvenile male and female mice [138]. In contrast, a limited bedding and nesting model of ELS increases PNN density in the basolateral amygdala, but only in juvenile male rats [45]. Meanwhile, exposure to an unpredictable chronic mild stressor (UCMS) paradigm does not result in significant alteration to PNN density during adolescence or adulthood in mice [93]. In humans, investigations into the impact of ELS on PNNs remain relatively limited. Still, available studies report either an increase in PNN density or no significant difference in adulthood following early life adversity [6, 131, 133]. Although findings across models and studies are not entirely consistent, they collectively suggest that PNNs are highly sensitive to the nature of ELS and exhibit region and sex-specific responses. These results highlight the complexity of PNN regulation in response to environmental stressors during critical developmental windows. The impacts of PNNs within neuropsychiatric disorders are complex and appear to be highly variable across studies, likely due to differences in stress models used to induce depressive- and anxious-like behaviors in rodent models. For example, in a study using the UCMS model to induce depressive-like behaviors in male adolescent rats, a 30-day incubation period was associated with both behavioral deficits and a decrease in PNN density and ACAN expression within the prelimbic cortex [151]. However, it is unclear if the reduction in PNN density is a causal factor in the emergence of depressive-like behaviors or a downstream consequence of the stress exposure. In contrast, a separate study utilizing an adult UCMS mouse model showed that chronic stress increased the transcription of astrocyte-derived ECM components, resulting in elevated PNN density. This increase was accompanied by heightened despair- and anxiety-like behaviors [122]. Furthermore, the enzymatic degradation of PNNs by intra-cortical injection of chABC reversed these depressive-like behaviors, suggesting that targeted remodeling of the ECM in adulthood may have therapeutic antidepressant-like effects [107]. These findings underscore the bidirectional and context-dependent relationship between PNNs and affective behaviors, and the need for further research to demonstrate their causal role in the pathophysiology of mood disorders.

### The role of PNNs within neurodevelopmental disorders

Disrupted maturation of neural circuits is a common hallmark of many neurodevelopmental disorders, and PNNs have emerged as key regulators of this process. Increasing evidence suggests that alterations in PNN formation, composition, or degradation are implicated in a broad range of neurodevelopmental disorders, including autism spectrum disorder, Down syndrome, and schizophrenia. Recent research has both explored PNNs across neurodevelopmental disorders (Table 2) and highlighted the need for further research into PNNs as therapeutic targets.

**Table 2:** PNN alterations in neurodevelopmental disorders and animal models

Disorder	Model	PNN change	Affected region	Physiological and behavioral consequence	References
Autism spectrum disorder	Human	Decreased PV cells with PNNs	Globus pallidus	Potential cognitive, sensory and motor behavioral defects	[8]
	*Cntnap2* mouse	Increased PNNs	Prefrontal cortex	Sociability deficits, particularly in male mice	[41]
	*Mecp2* mouse	Increased PNNs	Primary visual cortex	Precocious maturation and deficient binocular visual function	[58]
	*En2* mouse	Increased intensity of PNNs	Hippocampus and somatosensory cortex	Altered whisker texture discrimination and decreased preference for social novelty	[77]
	*Shank3b* mouse	No change in PNNs	Medial prefrontal cortex, somatosensory cortex and striatum	No physiological or behavioral changes reported directly associated with PNNs	[36]
	Valproic acid mouse	No change in PNNs	Medial prefrontal cortex, and somatosensory cortex	No physiological or behavioral changes reported directly associated with PNNs	[62]
	*Fmr1* mouse	Decreased PNNs Reductions in intensity of PNNs	Auditory cortex and amygdala CA2 Hippocampus	Impaired tone-associated memory formation	[105]
Down syndrome	Mice	Expanded PNN coverage	Hippocampal stratum oriens	Inhibited neurite outgrowth associated with age-related cognitive impairment	[50]
Schizophrenia	Human	Decreased PNNs	Dorsolateral prefrontal cortex	Impaired γ-oscillations potentially resulting in cognitive impairments	[32]
	Human	Decreased PNNs	Inferior colliculus	Potentially impaired frequency discrimination, deviance detection, and prepulse inhibition of the startle response	[56]
	Human	Decreased PNNs	Prefrontal cortex	Potential cognitive impairments, emotional dysregulation, psychotic symptoms, and persistent plasticity	[78]
	Human	Decreased PNNs	Lateral nucleus of the amygdala and lateral entorhinal cortex	Potential emotional dysregulation, memory deficits, social dysfunction, and psychosis	[97]
	Human	Decreased PNNs	Amygdala	Potential emotional dysregulation, impaired fear processing, mood instability, and social cognition deficits	[95]
	chABC Degraded PNN mouse	Decreased PNNs	Ventral hippocampus	Neuronal hyperexcitability, potentially associated with positive symptoms	[120]
	GCLM KO6^2^ KO mouse	Decreased PNNs	Thalamic reticular nucleus	reduction in spike bursting, indicating functional impairment in neuronal excitability	[124]

ASD is a broad class of neurodevelopmental disorders that is associated with different abilities in social interaction and communication, restricted interests, repetitive behaviors, and intellectual disabilities [60, 72, 108]. ASD has been previously associated with disruptions in cortical interneuron development across various genetic models [54, 57, 143, 150]. However, PNNs have been discovered to have bidirectional disruptions across various genetic models of ASD, suggesting an overall reduction in synaptic pruning and cognitive adaptation. Within a *Cntnap2* knockout mouse model of ASD PNNs enwrapping PV-positive neurons were found to be overexpressed in adults, suggesting a lack of synaptic plasticity within the model and accelerated maturation of PV + neurons [41]. Similar findings have been reported in the visual cortex of *Mecp2* knockout models and in the hippocampus and somatosensory cortex of *En2* knockout models [58, 77]. Within *Shank3b* knockout and Valproic acid models of ASD, reductions in PV expression were observed, but no changes in the densities of PNN-enwrapped PV-positive neurons were noted [36, 62]. Whereas within an *Fmr1* knockout, reductions in PNN density were found in the auditory cortex and amygdala, and reductions in the intensity of PNNs were found in the CA2 hippocampus [105]. Postmortem human studies of ASD are minimal, but have found the density of PV-positive neurons with a PNN significantly reduced in the globus pallidus [8]. The bidirectional relationship of PNNs across various models of ASD is likely due to the heterogeneity of this class of disorders and extreme genetic variability within ASD [76]. However, the variability impairments in PNNs highlight their complex role and importance in maintaining the balance of excitation and inhibition within ASD.

Manipulations of PNNs with ASD models are an emerging field of study. However, degradation of PNNs in models of ASD with upregulated PNNs showing ameliorated social deficits within the PFC and rescued plasticity with the hippocampus [16, 41, 69]. Whereas the genetic reduction of MMP-9 within an *Fmr1* knockout model of ASD has been shown to promote the formation of PNNs and normalize sensory deficits [144]. These limited results highlight the potential of PNNs as a therapeutic target for ASD. Degradation of PNNs may enhance synaptic plasticity, thereby facilitating greater adaptation in social behaviors and cognitive rigidity in individuals with ASD. However, limiting the degradation of PNNs may also be essential for proper circuit formation in specific models of ASD. It is crucial to note that these findings are limited, as they are restricted to a small number of genetic and pharmacological rodent models of ASD. Due to the high heterogeneity of ASD and inconsistencies between rodent and postmortem studies, further research is needed in both the morphology and degradation of PNNs in ASD.

Down syndrome (DS) is widely regarded as the most common genetic cause of intellectual disability, resulting from trisomy of human chromosome 21 [15]. Previous research has linked disruptions in synaptic structure, specifically within the hippocampus, with cognitive impairments associated with the Ts65Dn mouse model of DS [100]. A potential mechanism underlying synaptic structure disruptions may be the formation of PNNs. Gene expression studies have shown that chromosome 21 genes *Adamts1* and *Adamts5* are both triplicated in DS patients [71]. *Adamts1* and *Adamts5* encode for the enzymes ADAMTS1 and ADAMTS5, respectively, that degrade various lectins found in PNNs [52, 79]. Within a Ts65Dn mouse model of DS, this elevation of *Adamts1* and *Adamts5* was interestingly found not to reduce the number of PNNs. Instead, it resulted in expanded PNN coverage of neurons and a significant increase in VCAN2 within the hippocampal stratum oriens, associated with cognitive impairments. These results indicate a disrupted restructuring of PNNs with adult models of DS, which was associated with destabilized synaptic signaling [50]. This is further supported by previous findings showing that VCAN2 inhibits neurite outgrowth [117].

Despite the usual diffusion of PNNs within DS, application of chABC may prove to be an effective intervention. Due to the association between an increase in levels of VCAN2 and cognitive impairment, degradation of this lectin may prove to be an effective treatment. However, nearly all DS patients exhibit early-onset Alzheimer’s disease pathology, which is associated with disruptions in PNN integrity and abundance [113, 142]. Therefore, further degradation of PNNs via chABC may prove to be highly detrimental in the long run for DS patients. Extensive research is needed to further understand the complex role of PNNs within DS and potentially into the specific degradation of VCAN2.

Schizophrenia (SZ) is a complex developmental disorder that is characterized by symptoms that impact a patient’s perceptions, speech, cognition, behaviors, and emotions [118]. Despite the onset of symptoms of SZ beginning in late adolescence or early adulthood, SZ is considered a neurodevelopmental disorder due to the presence of cognitive and motor abnormalities before the onset of symptoms and a lack of neurodegeneration in post-mortem studies [92, 118]. Although research into the exact mechanisms of the pathogenesis of SZ is still ongoing, numerous postmortem studies have found notable decreases in PNNs within the entorhinal cortex, PFC, thalamic reticular nucleus, inferior colliculus and amygdala, with these findings being robust to confounding variables such as age of onset and pharmacological agents [32, 56, 78, 95, 96, 124]. However, unlike in models of ASD, the number of PV-positive interneurons was unchanged, suggesting that the decrease in PNNs does not merely reflect a loss of PV-positive interneurons. Furthermore, various genetic studies have shown that multiple genes associated with SZ encode for PNN components and related degrading enzymes [115, 116]. 

As described previously, PNNs serve a pivotal role in ion homeostasis and neuronal protection, with disruptions in these processes potentially linked to SZ pathology. PV-positive interneurons establish normal gamma oscillations and synchrony within cortical populations, which are essential for higher-order processing that is impaired in SZ [137]. Reductions in PNN formation around PV-positive interneurons may result in a lack of a proper cation buffer, leading to deficits in nerve conduction and neuronal hyperexcitability observed in SZ patients [5, 61]. Further studies have found that the enzymatic degradation of PNNs and exposure to DNA-alkylating agents in the ventral hippocampus induce dopamine system hyperfunction, a phenomenon previously theorized to underlie the positive symptoms of SZ [51, 120]. These findings are attributed to PV-positive interneurons lacking adequate protection due to reductions in PNNs, which may result in hyperactivity in dopaminergic circuits. Furthermore, multiple studies have reported elevated levels of MMP-9 in SZ patients; however, there is no evidence that this effect is seen before the onset of symptoms [30, 119].

However, despite clear evidence of disruptions in postmortem SZ studies and promising theories on the potential role of PNNs within the pathology of SZ, there is a lack of definitive research. Furthermore, due to the late onset of SZ symptoms, direct therapeutic manipulations of PNNs would be difficult. Currently, it is unclear if the application of MMP-9 inhibitors at the onset of known symptoms would be practical in rescuing reductions in PNNs. Notably, recent research has demonstrated that elevated plasma MMP-9 levels are associated with poor antipsychotic treatment response and deficits in white matter density in patients with SZ, suggesting that MMP-9 inhibition may be an effective therapeutic approach [66]. Further research into the electrophysiology of circuits related to SZ and direct manipulation of PNNs is needed to elucidate further the role of PNNs within the pathology of SZ and potential therapeutic treatments.

## Concluding remarks

PNNs play a critical role in maintaining neuronal stability while simultaneously regulating plasticity within mature neural circuits. Their complex molecular composition enables precise regulation of synaptic remodeling, neuronal protection, and experience-dependent plasticity in the brain. The spatial and temporal dynamics of PNN development highlight their regionally specific functions, which span a broad range of neural processes from memory consolidation to sensory and efferent signaling. These properties position PNNs as key modulators of both structural and functional plasticity across the lifespan.

Traditionally viewed as static barriers in the adult brain, PNNs are now recognized as highly dynamic. They adapt in response to learning, dual rhythms, and environmental stimuli, reflecting their involvement in ongoing neural remodeling. This shift in understanding highlights their complex and multifaceted role in the brain. It underscores the need for further research into the molecular mechanisms that control the development and remodeling of PNNs. Specifically, a more profound knowledge is necessary for how factors such as age, sex, and environmental stimuli influence their contribution to brain health and disease.

Therapeutically, manipulation of PNNs holds considerable promise. Interventions that enzymatically degrade PNN components, such as chondroitin sulfate proteoglycans, have been previously shown to induce or reopen periods of heightened plasticity, allowing increased synaptogenesis. On the other hand, the application of endogenous enzyme inhibitors may also be an effective method for preventing the pathological degradation of PNNs. However, further research is needed to elucidate the complex and dynamic roles of PNNs across neuropsychiatric conditions. Improper application of PNN-specific therapeutics could result in severe and potentially long-lasting cognitive, social, and emotional impairments. Furthermore, research into potential lifestyle-based interventions is severely lacking within the existing literature and should be robustly explored in addition to more invasive therapies.

Ultimately, PNNs are far from being just structural components of the nervous system; instead, they are dynamic regulators of plasticity throughout neuronal development and disease. As the field advances, a deeper understanding of how genetic predispositions and life experiences shape PNN development, maturation, and function will be key. Such knowledge will open pathways to precise, targeted interventions designed to restore or enhance neural circuit flexibility in health and disease.

## Data Availability

No datasets were generated or analysed during the current study.
